# On the Medicinal Use of Cobweb

**Published:** 1809-12

**Authors:** W. Simmons

**Affiliations:** Surgeon, at Manchester


### *On the Medicinal Use of Cobweb.* By W. SIMMONS, Surgeon, at Manchester.

THE use of Cobweb, in the treatment of ague, has lately engaged the attention of several of your correspondents. Very early in my professional life, I had extensive opportunities of seeing it employed in that disease, and with success. In the year 1778, intermittent fever was very prevalent, and did not yield, as formerly, to the Peruvian bark. The 'Tasteless Ague Drop' then came into vogue, as an empirical remedy, which, on analysis, proved to be a preparation of white arsenic. Before the constituent ingredients of this compound were known, it was employed chiefly by the patients themselves, and medical assistance was seldom sought, except to relieve from the effects of an over-dose. At this period, the gentleman with whom I then resided, among other circumstances, gave trial to cobweb. His method was to make ten grains of the cobweb into a bolus with conserve of roses, and to exhibit this quantity three times a day. This practice was attended with success, even in cases where the bark had previously failed. Writing from memory, at such a distance of time, I can only relate the fact; the recollection of which has been kept fresh in my mind, by occasionally mentioning it in conversation. Here the ague is rarely met with, and is generally an imported disease.

From one case that occurred, the body of the spider would seem to be possessed of the property of preventing the return of an intermittent, as well as the web:—A miller, of the name of Clowes, of his own accord, swallowed a large spider in half a pint of warm ale; the second paroxysm was weakened by it, and by taking a second dose, during the next interval, the disease was cured.

---

### *Origin of Ophthalmia.*

Among the causes of Ophthalmia, assigned by practical writers, I do not recollect that they have enumerated the bare inspection of the eye, when in an inflamed state. Yet I have met with something like it, and what, to one less cautious in inducting, might be assumed as equivalent to proof. A gentleman, who is but recently recovered from an attack, informed me, that, without any previous indisposition of these organs, when looking into the eyes of a friend, then afflicted with ophthalmia, he was himself suddenly seized with this disease. He described the sensation to be 'like a stream of something subtle issuing from the infected eyes into his own;' and which caused him present uneasiness. Whether so produced, or not, the inflammation in his case was certainly violent, and to subdue it required powerful means of depletion.

In turning over Montaigne's Essays, a few months ago, I met with a passage, which may be thought applicable to the subject. It is taken from the English edition by Cotton, printed in 1685, and with it I shall conclude:

> 'Dum spectant oculi læsos læduntur et ipsi.'

> Ovid. Amor. c. 2.

> 'Viewing some eyes, eyes to be sore are brought.'

*Nov.* 8, 1809.

